# Distinct Disease Phases in Muscles of Facioscapulohumeral Dystrophy Patients Identified by MR Detected Fat Infiltration

**DOI:** 10.1371/journal.pone.0085416

**Published:** 2014-01-14

**Authors:** Barbara H. Janssen, Nicoline B. M. Voet, Christine I. Nabuurs, Hermien E. Kan, Jacky W. J. de Rooy, Alexander C. Geurts, George W. Padberg, Baziel G. M. van Engelen, Arend Heerschap

**Affiliations:** 1 Department of Radiology, Radboud University Medical Center, Nijmegen, The Netherlands; 2 Department of Rehabilitation, Radboud University Medical Center, Nijmegen, The Netherlands; 3 Department of Radiology, Leiden University Medical Centre, Leiden, The Netherlands; 4 Department of Neurology, Radboud University Medical Center, Nijmegen, The Netherlands; University of Ulm, Germany

## Abstract

Facioscapulohumeral muscular dystrophy (FSHD) is an untreatable disease, characterized by asymmetric progressive weakness of skeletal muscle with fatty infiltration. Although the main genetic defect has been uncovered, the downstream mechanisms causing FSHD are not understood. The objective of this study was to determine natural disease state and progression in muscles of FSHD patients and to establish diagnostic biomarkers by quantitative MRI of fat infiltration and phosphorylated metabolites. MRI was performed at 3T with dedicated coils on legs of 41 patients (28 men/13 women, age 34–76 years), of which eleven were re-examined after four months of usual care. Muscular fat fraction was determined with multi spin-echo and T1 weighted MRI, edema by TIRM and phosphorylated metabolites by 3D ^31^P MR spectroscopic imaging. Fat fractions were compared to clinical severity, muscle force, age, edema and phosphocreatine (PCr)/ATP. Longitudinal intramuscular fat fraction variation was analyzed by linear regression. Increased intramuscular fat correlated with age (p<0.05), FSHD severity score (p<0.0001), inversely with muscle strength (p<0.0001), and also occurred sub-clinically. Muscles were nearly dichotomously divided in those with high and with low fat fraction, with only 13% having an intermediate fat fraction. The intramuscular fat fraction along the muscle’s length, increased from proximal to distal. This fat gradient was the steepest for intermediate fat infiltrated muscles (0.07±0.01/cm, p<0.001). Leg muscles in this intermediate phase showed a decreased PCr/ATP (p<0.05) and the fastest increase in fatty infiltration over time (0.18±0.15/year, p<0.001), which correlated with initial edema (p<0.01), if present. Thus, in the MR assessment of fat infiltration as biomarker for diseased muscles, the intramuscular fat distribution needs to be taken into account. Our results indicate that healthy individual leg muscles become diseased by entering a progressive phase with distal fat infiltration and altered energy metabolite levels. Fat replacement then relatively rapidly spreads over the whole muscle.

## Introduction

Facioscapulohumeral muscular dystrophy (FSHD) is the third most common hereditary muscular disorder [1]. The disease is characterized by progressive asymmetric weakness and fatty infiltration of skeletal muscles. In recent years it was demonstrated that FSHD is associated with a contraction of D4Z4 repeats on chromosome 4q35 [2], leading to lost repression of DUX4, a protein that exerts toxic effects on muscle cells [3].

Even though the most important genetic event for the disease seems to be identified, a causative treatment is not yet available [4]. Progress is hampered because the trigger for DUX4 expression and the further unfolding of disease processes leading to fatty infiltration and muscle weakness are not known. Thus clarification of the underlying mechanisms is expected to offer clues for a more targeted approach in the search for treatment [5]. Understanding these mechanisms first requires that some key questions concerning the process of fatty infiltration are addressed. What is the natural distribution of fatty infiltration? How is this related to clinical severity, to muscle weakness and to energy metabolism? Is there prevalence for specific muscles to be affected and does fatty infiltration vary within muscles? What is the natural progression over time and what are predictive signs of progression?

To answer these questions and to evaluate treatment effectiveness, the use of a non-invasive quantitative imaging method, such as MRI, is essential. Unlike biopsies, MRI is not limited to a single location, and longitudinal data can be collected without risk for the patient. MR of fatty infiltration in muscles has been used to study muscular disorders like Duchenne muscular dystrophy [6], [7]. We have introduced a quantitative MRI measure of fatty infiltration in muscles based on T2 relaxation time analysis and demonstrated its value in a preliminary study of FSHD patients [8]. Phosphorus MR spectroscopy has been used extensively to investigate the energy status of diseased muscles [9]–[15]. Recently it was also introduced in a pilot study with FSHD patients [16].

Until now quantitative MR imaging studies were performed in limited numbers of patients. However, because of the variability in age of onset and in degree of disease severity [17], a study of its pathophysiology requires the participation of a relatively large number of well described patients. The main aim of this study was to determine natural disease state and progression by quantitative MRI of skeletal muscles in the legs of a large, well-characterized cohort of genetically confirmed FSHD patients. In particular we wanted to address the aforementioned pathophysiological questions to ultimately uncover clues on disease mechanism and to establish MRI biomarkers with prognostic and predictive value for personalized assessments.

## Materials and Methods

### Patients and Study Design

We recruited 41 FSHD patients from the local neurology department (28 men/13 women, age 21–81 years, see Table 1 for patients demographics). Of 36 patients the upper leg (‘thigh’) was examined, they were selected from a group of patients that were entering a clinical trial to assess the effects of rehabilitation intervention [18]. In addition we included the MR exams of the lower leg of five patients from a previous study [8], which were re-analyzed in the exact same way as the MR exams of the aforementioned patients (vide infra).

**Table 1:** Patient demographics.

Patient nr.	Ricci-score	Sex	Age (years)	FSHD duration (years)
1	0	f	21	15
2	1.5	f	25	2
3	1.5	m	31	5
4*	2	m	34	3
5*	3	m	34	9
6	1.5	m	38	17
7	2	m	38	16
8*	3	m	39	19
9	1	f	42	5
10	3.5	m	44	32
11*	2	m	48	14
12	4	m	49	40
13	3.5	m	50	14
14	1.5	f	50	1
15	1.5	f	51	6
16*	4	m	52	19
17*	3.5	m	53	9
18	4.5	f	54	24
19*	3	f	55	1
20*	1.5	m	55	21
21	2	f	56	2
22	2	f	56	22
23	3.5	m	57	17
24	4	m	57	41
25	4	m	58	41
26*	2	f	59	22
27	3	m	59	41
28	4	m	60	9
29	3	m	61	22
30	3.5	m	61	17
31	4	f	63	31
32	4	m	64	15
33	4	m	66	30
34	3	m	66	9
35*	4.5	f	66	49
36	3	m	66	6
37	3	f	68	12
38	2	m	69	na
39	3	m	69	19
40*	3.5	m	76	4
41	3.5	m	81	3

Underwent two MR exams four months apart.

na = not available.

Eleven patients (8 men/3 women, age 34–76 years) were randomly selected, from the group that underwent an MR examination of the thigh, for a follow-up measurement after a period of four months. During this period these patients were instructed to maintain a normal level of activity (‘usual care’).

All patients were clinically and genetically diagnosed with FSHD and able to walk independently (ankle-foot orthoses and canes were accepted). Patients were all unrelated except for one mother and son (patients #7 and #37). Disease severity was assessed with the Ricci score [19] and maximum voluntary isometric extension (quadriceps) and flexion (hamstrings) of the knee were measured with a quantitative fixed myometry testing system [20]. Ethical approval was obtained from the Radboud university medical center review board, and written informed consent was obtained from all subjects.

### MR Methods

MR measurements were performed on a 3T MR system (TIM Trio, Siemens, Erlangen, Germany). Subjects were positioned feet first supine inside the magnet bore. Images were acquired with a home-built proton birdcage radiofrequency coil (inner diameter 25 cm).

In 36 patients, the least affected thigh, according to the subject’s own experience was examined, unless there were contraindications (e.g. a previous fracture or recent injury). A fish oil capsule was positioned at one third of the distance between the spina iliaca anterior superior and the patella and served as a landmark for exact matching of the imaging slices between the baseline and follow-up measurements. For the MR examinations of leg, the upper end of the proton coil was positioned at the center of the patella.

#### MR imaging

Scout images were made in three orthogonal directions to position MRI slices for subsequent scans. The transmit frequency was set on the water resonance and the transmitter voltage was adjusted to the load.

All imaging was performed in the transversal plane centered on the middle femur for the thigh, or the largest circumference for the leg.

T1 weighted spin echo (SE) MR images were acquired first (field of view (FOV) 175×175 mm; base resolution 384; repetition time (TR) 530 ms; echo time (TE) 16 ms; slices 23; slice thickness 4 mm; gap 0.4 mm).

Turbo Inversion Recovery Magnitude (TIRM) images were collected with an inversion time (IT) to null the fat signals (FOV 175×175 mm; base resolution 256; TR 4000 ms; TE 41 ms; IT 22 ms; slices 23; slice thickness 4 mm; gap 0.4 mm) to visualize edema [21]–[23]. To avoid inflow artifacts from venous and arterial blood, saturation bands were placed above the upper and below the lower slice [24].

Subsequently, multi SE MR images were acquired (FOV 175×175 mm; base resolution 256; TR 3000 ms; 16 equally spaced TE’s 7.7–123.2 ms; slices 5–8, limited by specific absorption rate; slice thickness 6 mm; gap 9 mm).

#### Phosphorus MR spectroscopic imaging (^31^P MRSI)

A ^31^P quadrature insert surface coil covered the quadriceps muscles of the thigh, and for the leg measurements a circularly polarized half volume ^31^P coil covered the calf musculature. A 3D ^31^P MRSI dataset was acquired after imaging (FOV 150×150×200 mm; matrix-size 14×14×8 quadriceps/10×10×8 calf, TR 1000 ms; BIR45 adiabatic pulse for excitation; 12 averages; weighted k-space acquisition; nominal voxel volume 8.6 ml quadriceps/16.6 ml calf). Datasets were interpolated to a matrix size of 16×16×8.

### Data Analysis

#### MR imaging

**Figure 1:**
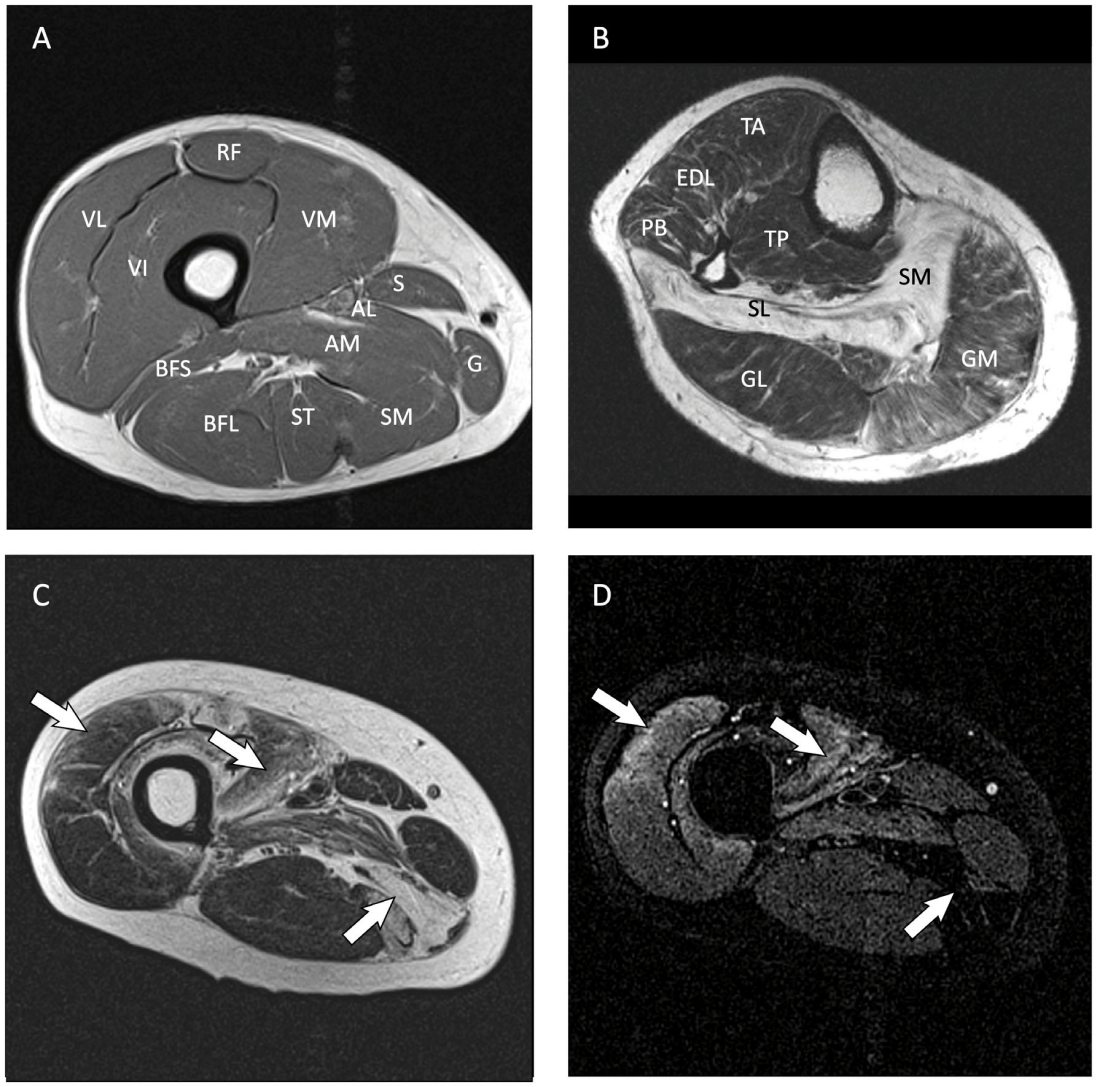
Typical transversal T1 weigthed and TIRM MR images of FSHD patients. (**A**) Transverse T1 weigthed image of the thigh of a male FSHD patient (age 38), showing fatty infiltration (hyperintense signal) in the semimembranosus and semitendinosus muscles. (**B**) Transverse T1 weighted image of the leg of a male FSHD patient (age 66 year). Fatty infiltration of the soleus muscles is clearly visible. (**C**) Transverse T1 weighted image of the thigh of a 39-year-old male FSHD patient. (**D**) Corresponding TIRM image. The semi-membranosus is clearly fat infiltrated (grey striped arrow), this results in a nulled signal on the corresponding TIRM image. In contrast, the vastus lateralis and vastus intermedius show hyperintense signal in the TIRM images (white arrows) reflecting edema or inflammation. The different muscles in the thigh (Fig. 1.A) and calf (Fig. 1.B) are indicated by the following abbreviations: rectus femoris (RF), vastus lateralis (VL), vastus intermedius (VI), vastus medialis (VM), sartorius (S), adductor longus (AL), adductor magnus (AM), gracillis (G), semi membranosus (SM), semi tendinosus (ST), biceps femoris long head (BFL) and biceps femoris short head (BFS), tibialis anterior (TA), extensor digitorum, longus (EDL), peroneus brevis (PB), tibialis posterior (TP), soleus medialis (SOM), soleus lateralis (SL), gastrocnemius medialis (GM) and gastrocnemius lateralis (GL).

Each of the investigated muscles (see Fig. 1) was analyzed separately. T1weighted images were scored for fatty infiltration using the four grade scale of Lamminen [25], by one experienced musculoskeletal radiologist (J.W.J.R). When a different score was awarded to the proximal and distal images the average score was used.

Muscle area was assessed by drawing regions of interest (ROI’s) for every muscle on the center slice of the T2weighted MRI. Fat and muscle fractions were quantified from the multi SE MR images as described earlier [8]. Note that normal fat fraction for healthy muscle does not exceed 10% [26]. This method is not suitable when edema is present, as it will affect the tissues transverse relaxation properties. In those cases, T1 signal intensity (SI) and TIRM SI of the individual muscles were quantified by carefully drawing ROI’s in ImageJ (http://rsb.info.nih.gov/ij/) and normalized to bone marrow SI.

To assess natural progression fat fraction differences were normalized to a period of one year for every patient (for every muscle) by dividing these fractions by the exact number of days between the baseline and follow up measurement, multiplied by 365.25.

#### ^31^P MRSI

From the middle slice of the 3D-MRSI dataset with the largest circumference, representative voxels were assigned to a specific muscle according to the corresponding T1weighted image overlaid with the MRSI grid. Only spectra with a sufficient signal-to-noise-ratio (SNR) (Cramer Rao Lower Bound (γATP) <30%) were included for further analysis.

Free induction decays were zero-filled to double the number of points and apodized by 8 Hz with a Lorentzian line shape and manually phased using jMRUI 4.0 [27]. Peak areas were obtained from inorganic phosphate (Pi), PCr (fitted to a Lorentzian line shape), and ATP (fitted to a Gaussian line shape), using the AMARES algorithm [28] with prior knowledge on the relative line width, frequency and amplitude.

Metabolite ratios: PCr/ATP and Pi/ATP were evaluated to avoid coil profile variations. The pH was calculated from the Pi-PCr frequency shift [29]. The value of each parameter was averaged for all analyzed voxels in one muscle and this value was used for further analysis.

### Statistics

Statistical analyses were performed with Prism 5.0 (GraphPad Software, San Diego, California, USA). Non-parametric one-way-ANOVA (Kruskal-Wallis test) was used to investigate differences in the average fat fraction between muscles, with Dunn’s Multiple Comparison Test as post-hoc test. One-tailed correlation analyses were performed between fat fraction and patients’ age, duration of disease, radiological score, Ricci-scores, maximum voluntary force, PCr/ATP, Pi/ATP, and pH. Linear regression analysis was used to assess the distribution of fatty infiltration over the length of the muscle. Outcome parameters in this analyses are the slope of the line, indicating the direction of fatty infiltration over the length of the muscle, and the coefficient of determination (R^2^), indicating to what extent fat fraction increases or decreases linearly over the length of the muscle. One-way ANOVA was used to investigate dependence of fat fraction progression on initial muscle fraction. T1 SI difference was compared between muscles normal and hyperintense TIRM images with a one-tailed t-test, and correlation was investigated with linear regression.

## Results

### Muscular Fat Infiltration, Edema, Clinical Grading and Muscle Strength in FSHD

Fat infiltration in skeletal muscles is visible as hyperintense areas on T1 weighted MR images (Fig. 1A–C). This may be accompanied by edema, which can be identified independently from fatty infiltration by TIRM images (Fig. 1D). In 41 FSHD patients we investigated 446 leg muscles, of which 4.3% showed edema, which was mostly present in the quadriceps muscles.

**Figure 2:**
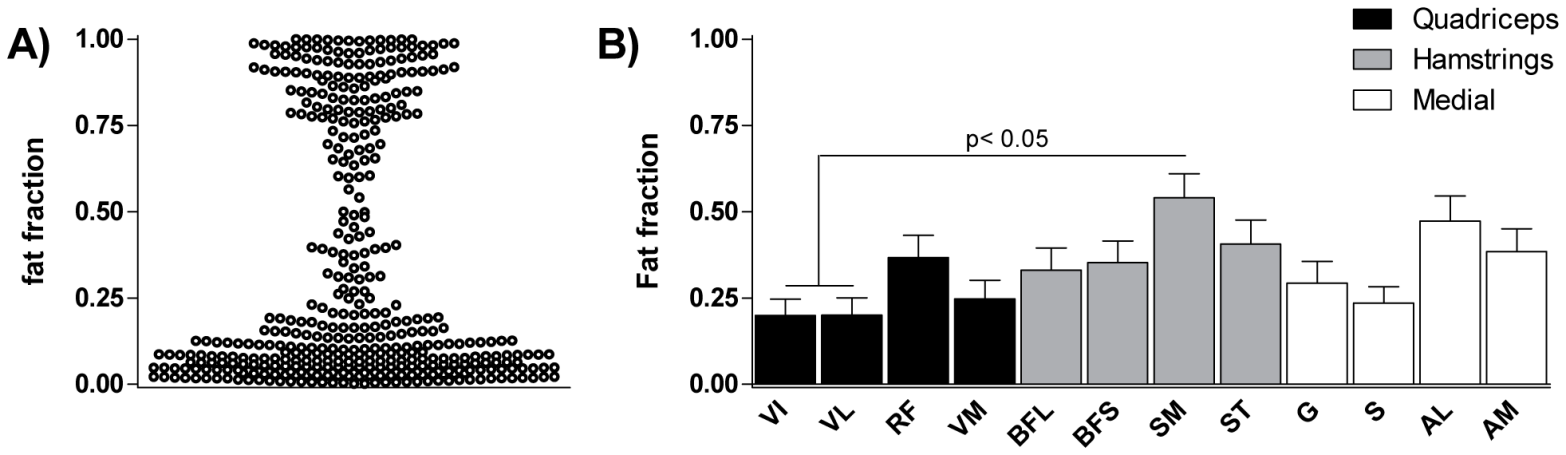
Distribution of naturally occuring fat fraction of the thigh muscles of a large cohort of FSHD patients. (**A**) Fat fraction distribution over all muscles. Fat fraction of 0 signifies 100% muscle, 1 indicates 100% fat. Muscles with an intermediary fat fraction (>0.25 and <0.75) are observed, in ∼13% of the investigated muscles. (**B**) Involvement of individual thigh muscles in FSHD. Average fat fraction of 36 patients. Error bars (SEM) reflect the high variability in this fraction between patients. The SM appears to be the most affected muscle of the upper leg, having a significantly higher average fat fraction (0.54±0.41) compared to the VL or VI (0.20±0.29, 0.20±0.27, respectively). Note that fat fractions are not Gaussian distributed therefore reporting only mean±error values is not a good representation of the data.

The quantitative assessment of muscular fat fraction revealed that 262 of the remaining 427 muscles were normal or mildly fat infiltrated (<0.25 fat fraction), 54 were intermediately fat infiltrated (between 0.25 and 0.75 fat fraction) and 111 muscles were severely infiltrated (>0.75 fat fraction). A fat fraction distribution plot resulted in a typical hourglass shape (Fig. 2A). Significant differences were observed in average fat fractions of thigh muscles (p<0.01), in particular the semimembranosus had a significant higher fat fraction than the vastus lateralis and vastus medialis (Fig. 2B).

**Figure 3:**
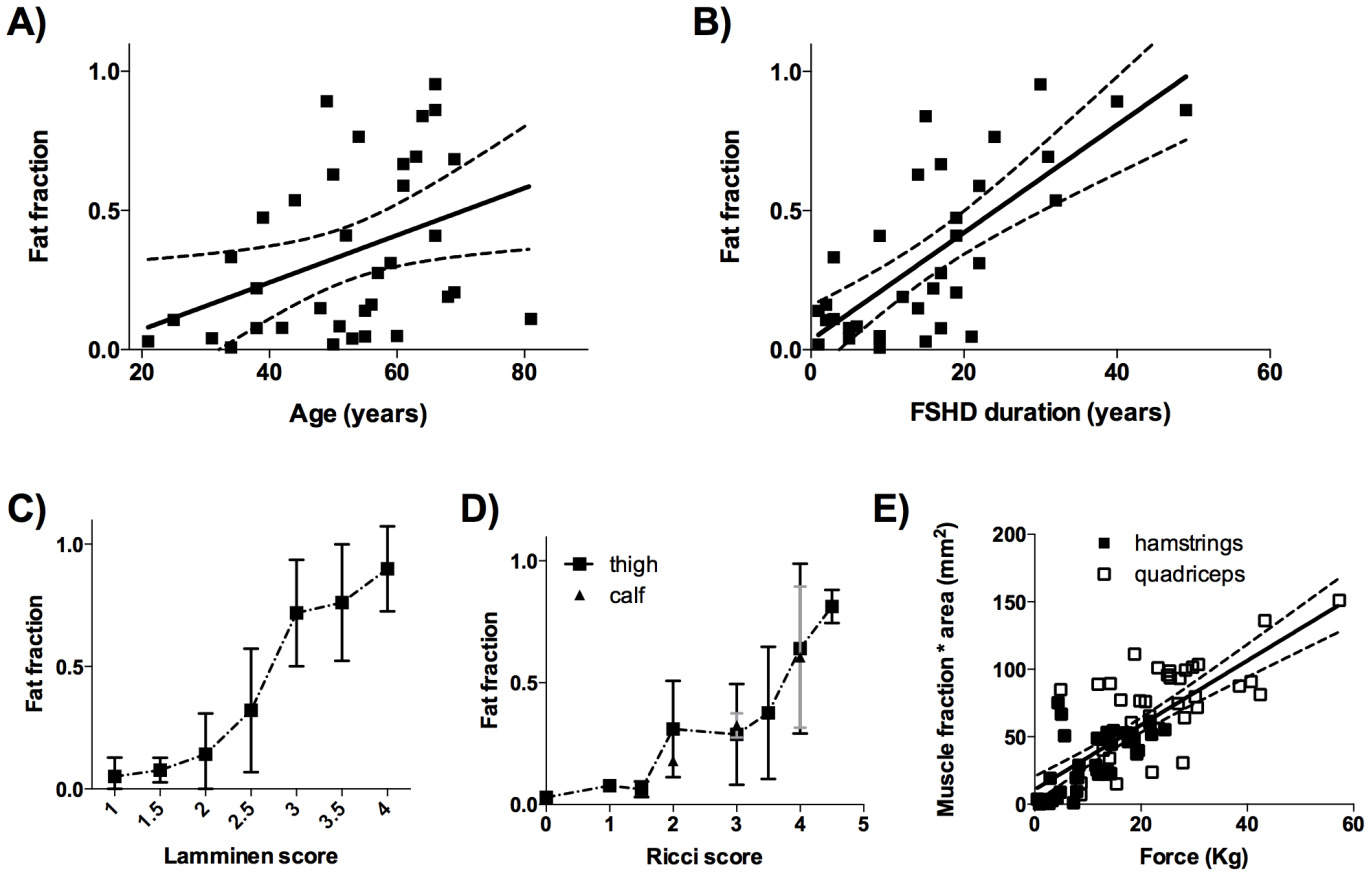
Correlation of fat or muscle fraction, determined by quantitative MRI, with clinical scores. (**A**) Correlation between age of the patient and average fat fraction of the thigh (p<0.05, R^2^ = 0.15). (**B**) Average fat fraction of the thigh and FSHD duration are highly correlated (p<0.0001, R^2^ = 0.54). (**C**) Fat fraction highly correlates with the radiological Lamminen score of the corresponding muscle (p<0.0001, R^2^ = 0.70). (**D**) Quantitative fat fraction of lower limb correlates with patients Ricci score (p<0.0001, R^2^ = 0.90). Fat fraction starts to increase above normal levels at Ricci score 2. The high standard deviation depicted in the error bars signifies the large variation in fat fraction determined in the limb and the appointed Ricci score. (**E**) Correlation between muscle fraction (1-fat fraction) and force of quadriceps and hamstring muscle groups (p<0.0001 and R^2^ = 0.76).

Average fat fraction correlated positively with patients’ age (p<0.05, R^2^ = 0.15) (Fig. 3A) and FSHD duration (p<0.0001, R^2^ = 0.54), (Fig. 3B). Slopes of the correlations were not significantly different between muscles (Fig. S1 and S2). The average yearly increase in fatty infiltration was 0.8±0.4% for age and 1.9±0.3% for FSHD duration. The average fat fraction showed a strong correlation with radiological scores (p<0.0001, R^2^ = 0.70) (Fig. 3C) and with overall clinical Ricci score for FSHD severity (p<0.0001, R^2^ = 0.90) (Fig. 3D). The fat fraction deviated from normal at Ricci score 2 (a subclinical event as this score excludes leg muscle involvement) and further increased at higher Ricci scores. Muscle fraction multiplied by the muscle area significantly correlated with muscle strength for the quadriceps and hamstring (p<0.0001, R^2^ = 0.57) (Fig. 3E).

### Intramuscular Fat Distribution

**Figure 4:**
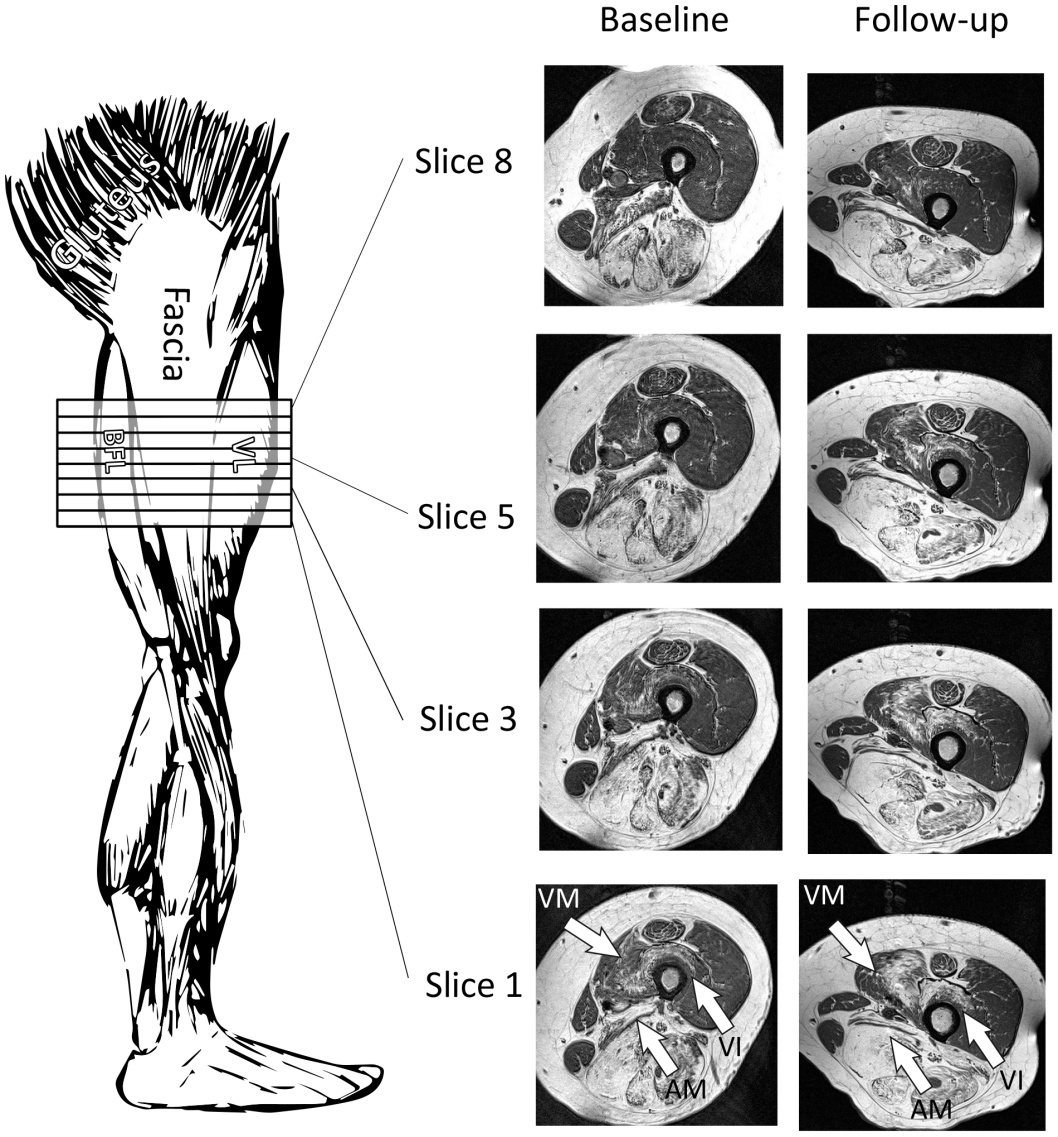
Intramuscular distribution and progression of fatty infiltration. Transveral T1 weighted images at different positions of the thigh of a FSHD patient. Baseline measurement (left panels) reveals an uneven distruibution over the length of the muscle with an increasing fat infiltration from proximal (top) to distal (bottom), especially prominent in the VM, VI, AM. This fatty gradient was largest in intermediate fat infiltrated muscles, as was shown by the linear regression analyses. These intermediately fat infiltrated muscles also showed the largest increase in fatty infiltration over time. From the follow-up measurement (right panels) it is clear to see that fat is increasing distally. AM = adductor magnus; BFL = biceps femoris long head; VI = vastus intermedius; VL = vastus lateris; VM = vastus medialis.

Visual inspection of MR images revealed that the fat fraction was often not evenly distributed over the length of the muscle (Fig. 4). Muscle with an intermediate fat fraction showed the steepest fatty infiltration gradient over the length of the muscle (7±1% cm^−1^, mean±SEM). This value was significantly higher compared to muscles that were normal or mildly fat infiltrated (1.3±0.3% cm^−1^, p<0.0001) and those that were heavily infiltrated by fat (1.1±0.1% cm^−1^, p<0.0001). Overall fat fraction rose from proximal to distal.

### Natural Progression of Fatty Infiltration

The natural progression of fatty infiltration was investigated for 85 muscles of eleven patients. An average increase in fat fraction of 0.054±0.12 per year was observed. In intermediately affected muscles (n = 12) the progression of fatty infiltration was much faster (0.18±0.15 per year) as compared to heavily fat infiltrated muscles (0.00±0.10 per year, n = 20) and to normal to mildly infiltrated muscles (0.043±0.10 per year, n = 53). Natural progression in fat infiltration depended on the initial muscle fraction (p<0.01) and appeared to increase from distal to proximal (Fig. 4).

**Figure 5:**
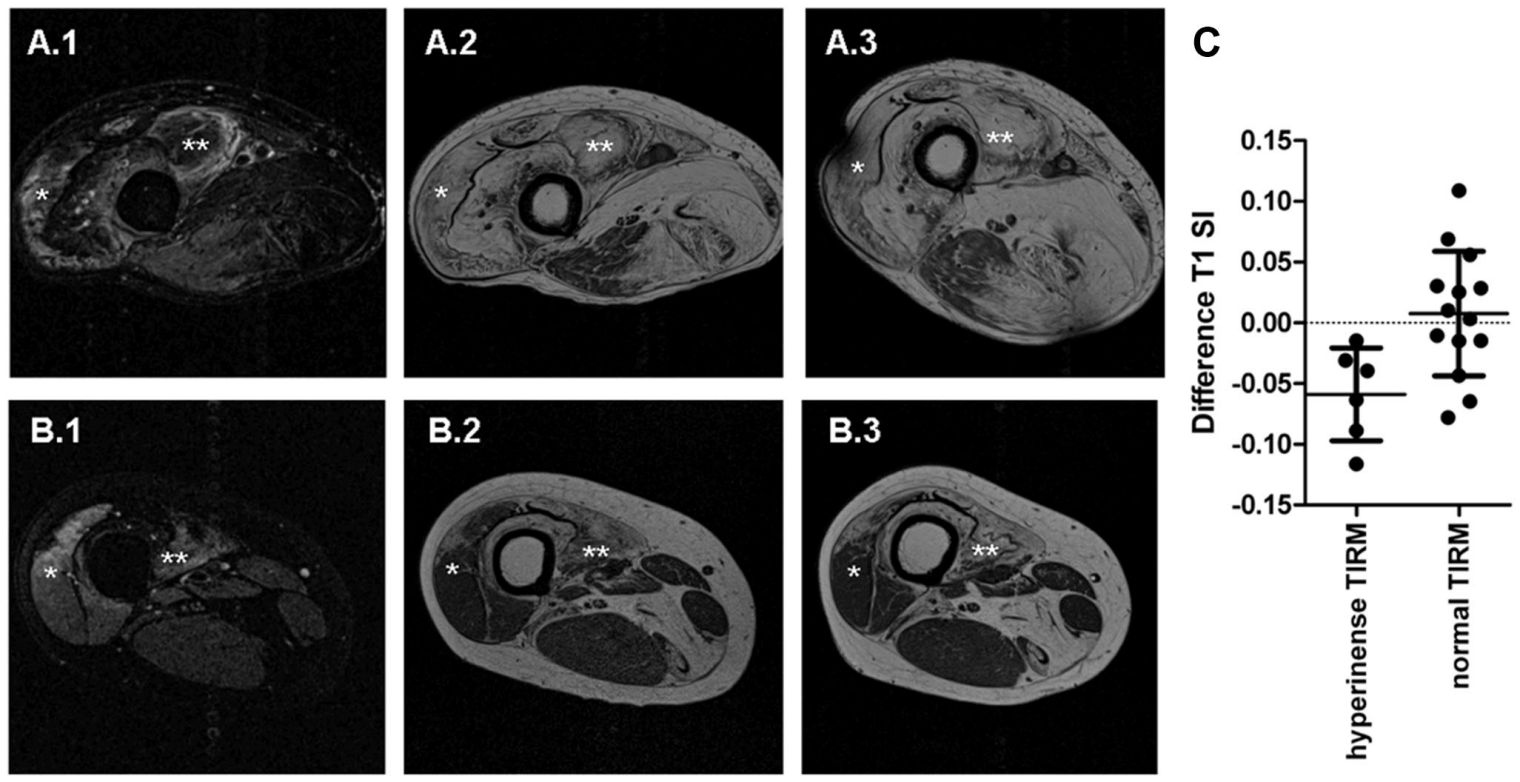
The presence of edema, as identified by TIRM imaging, correlates with increased fatty infiltration, as reflected in changes in T1 weighted images. (**A**) TIRM and T1 weighted images of a 76 year-old male FSHD patient. (**B**) TIRM and T1 weighted images of a 39 year-old male FSHD patient. (**A–B.1**) VL(*) and VM(**) muscles of two FSHD patients show hyperintensity on TIRM images, indicating edema. (**A–B.2**) Baseline T1 weighted images. (**A–B.3**) Follow-up T1 weighted images showing an increase of fatty infiltration after about 4 months in the VL(*) and VM(**) muscles. (**C)** SI difference between baseline and follow-up T1 weighted images is significantly different in TIRM hyperintense FSHD muscles (N = 6) compared to TIRM normal FSHD muscles (n = 14) (p<0.01).

Six muscles, in two patients, showed hyperintensity on the baseline TIRM images, indicating edema. The T1 SI difference between baseline and follow up exam, representing fat infiltration, was significantly different in muscles with hyperintense signal on baseline TIRM images compared to TIRM normal muscles (n = 14) of the same patients (p<0.01) (Fig. 5). Linear regression analysis showed a trend between the TIRM SI and the difference in T1 SI (p<0.1, R^2^ = 0.1).

### High-energy Phosphate Metabolites

**Figure 6:**
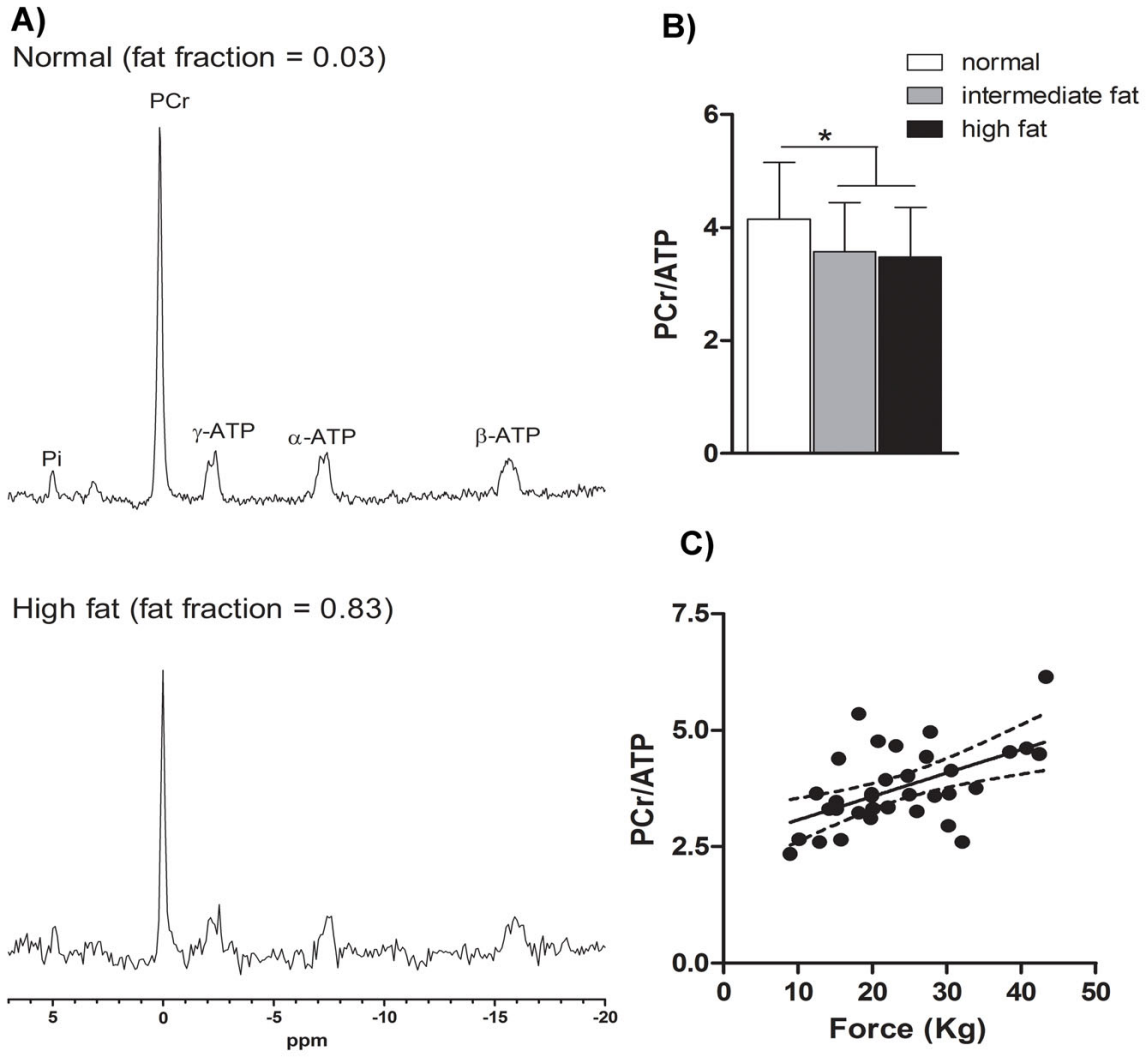
High-energy phosphates in the different stages of fatty infiltration and correlation with muscle force. (**A**) Representative phosphorous MR spectra of VL muscle of FSHD patients, upper with a normal fat fraction, lower with a high fat fraction. (**B**) PCr/ATP decreases with fat fraction (mean±SD). In intermediately fat infiltrated muscles the PCr/ATP is already decreased significantly from 4.15±1.00 to 3.57±0.88. Completely fat infiltrated muscles do not show a further decrease of this ratio. (**C**) Significant correlation between PCr/ATP and muscle strength (p<0.001, R^2^ = 0.29). Pi = inorganic phosphate; PCr = phosphocreatine; ATP = adenosine triphosphate.

Analysis of phosphate metabolites by ^31^P MRS revealed that the PCr/ATP ratio correlated with the fat fraction of the specific muscle (quadriceps p<0.05, R^2^ = 0.06, calf p<0.01, R^2^ = 0.33). This PCr/ATP ratio is significantly decreased in the intermediately fat infiltrated muscles compared to muscles with a normal fat fraction (p<0.05), but was not further decreased in muscles with a high fat fraction (Fig. 6A). The PCr/ATP ratio also correlated with muscle force (p<0.001, Fig. 6B).

## Discussion

In this study we identified three distinct phases of fat infiltration in lower limb muscles of FSHD patients by quantitative MR. An analysis of the average fat fraction for all individual muscles uncovered an hourglass pattern of many muscles with either very low or high fat, and few muscles with an intermediate fat fraction. This quasi-binary distribution has not been reported for other muscular dystrophies [7] and may be FSHD specific. The intermediate phase is most characteristic, showing a relative steep fat gradient over the length of the muscles, an altered energy metabolism and rapid progression of fatty infiltration.

For other dystrophies often average values of all subjects or muscles are presented, which obscures the presence of a specific distribution. The average fat fraction as calculated over all investigated muscles in this study (0.3±0.1), actually only was present in 36 out of 427 muscles. Fat fractions were highest in the semi membranosus, semi tendinosus and adductor muscles as has been previously described by Wattjes et al. [21]. The vastus muscles were largely preserved.

We found that in leg muscles the intramuscular fat fraction increased linearly from proximal to distal, as was also observed in a pilot study of only the lower leg [8]. The steepest fat gradient occurred in the intermediate affected muscles indicating that these muscles are progressing towards a complete fat infiltrated state. This interpretation is supported by the follow-up measurements, which revealed that intermediate affected muscles were most prone to increase their fat-muscle ratio. In these muscles the average increase of this ratio was about 10% in four months. This may seem fast for a disease that is characterized by slow progression, but we observed it in only a relative small fraction of muscles. The quasi-binary fat distribution of muscles in FSHD patients mentioned above also indicates that relative rapid transitions occur. Moreover, a sudden disease progression within individual muscles is in accordance with the often reported observation in FSHD patients of periods of rapid deterioration of single muscles or muscle groups, interrupting long stable periods [30], [31]. In some cases the lower performance of a single muscle may be compensated by unaffected synergistic muscles, which would clinically mask its dysfunction [32], [33]. Assuming that replacement of muscular tissue by fat occurs at a constant rate after entering this intermediate phase, fat replacement of entire muscles will, on the average, be completed within approximately three and a half years. This can be relevant for prognostication and monitoring therapy effectiveness in FSHD. There is no report on fat gradients over the length of muscles in other neuromuscular disorders, which may be FSHD specific. Recently, a muscular fatty content gradient was also found in inherited poly-neuropathy, but this was not associated with disease progression [34].

The low percentage of muscles involved in a rapid progression towards complete fatty infiltration indicates that this process is triggered by an infrequent event. The nature of this event is unknown, but the initial relatively high distal level of fatty infiltration and the differences between muscles suggests a local origin. This is in agreement with findings that only 0.1% of muscle nuclei express DUX4 in FSHD patients [35]. A recent paper by Tassin and colleagues [36] describes a model of initiation and propagation of a transcriptional cascade, which provides an elegant explanation for our observation of a gradient of fatty infiltration and fast progression in intermediate fat infiltrated muscles. In this model the activation of the DUX4 gene in (one or) few myonuclei yields DUX4 protein molecules that diffuse into the cytoplasm towards neighboring nuclei where they activate target genes, which causes expansion into a transcriptional cascade of deregulation. Because of the multinucleated nature of myofibers this model predicts a gradient of deregulation over the length of muscles. The amplification of DUX4 gene activation into a transcriptional cascade may also explain the fast progression observed in the intermediate fat infiltrated muscles. Preferential involvement of particular muscles (e.g. semi membranosus) and distal initiation may hide clues towards the initial DUX4 gene activation trigger. Our finding that MR-visible fat content increases more in the case of initial edema supports the involvement of inflammation in early disease onset, as TIRM positive muscles are associated with muscle inflammation [21]–[23], [37]–[39]. However, whether inflammation is cause or consequence of DUX4 transcription in the initiation process remains unclear.

The correlation between increased fat fraction and lower strength of skeletal muscles is coherent with the loss of muscle mass and also explains the (weak) relation with the age of the patients. Clinical severity scores (Lamminen [25] and Ricci [19]) strongly correlated with fat fraction, but abnormal high fat fractions were also present in lower limb muscles without clinical symptoms, as was observed in patients with Ricci score 2 (excludes lower limb involvement). Thus, imaging fatty infiltration is a potential tool to predict clinical muscle affliction [32], [33]. The extent of edema in our study (4.3%) is somewhat lower than reported in two recent FSHD studies, that however, included more muscles per patient and more severely affected patients [23], [40].

The lower PCr/ATP ratios observed in intermediately fatty infiltrated muscles suggest an early change in high-energy phosphate metabolism in disease development. Lower steady state PCr/ATP ratios were also found in muscles of Becker and Duchenne patients [10], [41]. This may represent a lower cellular (phospho)creatine pool due to a lower energy state. Alternatively, it may represent a change in fiber type composition, if the fraction of oxidative fibers, which have lower PCr/ATP ratio’s [42], [43], increase due to preferred involvement of type II fibers. This is supported by histological findings of biopsies, showing more dominant type I fibers among the remaining fibers in FSHD affected muscles [44] and is also congruent with the correlation between PCr/ATP and muscle force.

Taking muscle biopsies remains the gold standard to examine muscular dystrophies, but this is invasive, painful, restricted to a limited number of biopsy sites and only provides focal information. As observed in the present and a previous study [8] fatty infiltration is very heterogeneous, both between and within muscles, which demonstrates the need to know in advance which (part of a) muscle is affected, to acquire representative tissue. Our study indicates that MRI guidance in taking muscle biopsies is needed. Other common imaging techniques have disadvantages, such as radiation exposure in computer tomography, or poor signal to noise and limited penetration depth in ultrasound. In clinical trials muscle strength is often assessed to evaluate treatment effects, but this may show a placebo effect [45]. Muscular fat fraction determined by MRI does not involve a placebo effect.

A limitation of our study was the lack of including a component for the presence of edema in the T2 analysis. However, we identified muscles with edema by TIRM and excluded the very small fraction of edematous muscles from this T2 analysis. Progression of fatty infiltration in these muscles was then derived from T1 images. Furthermore, we chose to investigate lower extremity muscles in these patients even though FSHD is a disease known to first involve the facial and scapular muscles. However, for this study we aimed for the highest image quality, which could be achieved with a dedicated coil for the lower extremity. To compare different disease phases we had to introduce fat fraction cut-off values, for which we chose 25% and 75% of fatty infiltration. Shifting these values by ±5% did not change the main results of this study.

In conclusion, this study established fat fraction as assessed by MR imaging as an objective quantitative and sensitive biomarker for muscular affliction in FSHD, detecting even subclinical muscle involvement. This MR biomarker may serve to predict disease progression, to guide biopsies and to evaluate treatments to preserve or improve muscle performance. Importantly, in these applications the intramuscular fat distribution may have to be taken into account. Our data suggest a specific sequence of events that leads towards full muscle pathology in FSHD, in which muscles first progress from normal to being distally fat infiltrated, with an altered metabolic profile, after which fat rapidly infiltrates the whole muscles. This process of disease unfolding may direct new treatment strategies.

## Supporting Information

Figure S1 **Correlation between fat fractions and age for the individual thigh muscles.** Solid line gives best linear correlation with 95% confidence interval indicated by the dotted lines. Slopes of the lines were statistically tested to identify possible differences between the muscles. However analyses showed no significant differences. VL = vastus lateralis, VI = vastus intermedius, RF = rectus femoris, VM = vastus medialis, BFS biceps femoris short head, BFL = biceps femoris long head, S = sartorius, G = gracillis, ST = semit endinosus, SM = semi membranosus, AM = adductor magnus, AL = adductor longus.
(TIF)

Figure S2 **Correlation between fat fractions and disease duration for the individual thigh muscles.** Solid line gives best linear correlation with 95% confidence interval indicated by the dotted lines. Slopes of the lines were statistically tested to identify possible differences between the muscles. However analyses showed no significant differences. VL = vastus lateralis, VI = vastus intermedius, RF = rectus femoris, VM = vastus medialis, BFS biceps femoris short head, BFL = biceps femoris long head, S = sartorius, G = gracillis, ST = semi tendinosus, SM = semi membranosus, AM = adductor magnus, AL = adductor longus.
(TIF)
