# Supplementary figures and images for: Distinct Disease Phases in Muscles of Facioscapulohumeral Dystrophy Patients Identified by MR Detected Fat Infiltration
Source: PLoS One. 2014 Jan 14;9(1):e85416. doi: 10.1371/journal.pone.0085416 (PMC3891814; doi:10.1371/journal.pone.0085416)

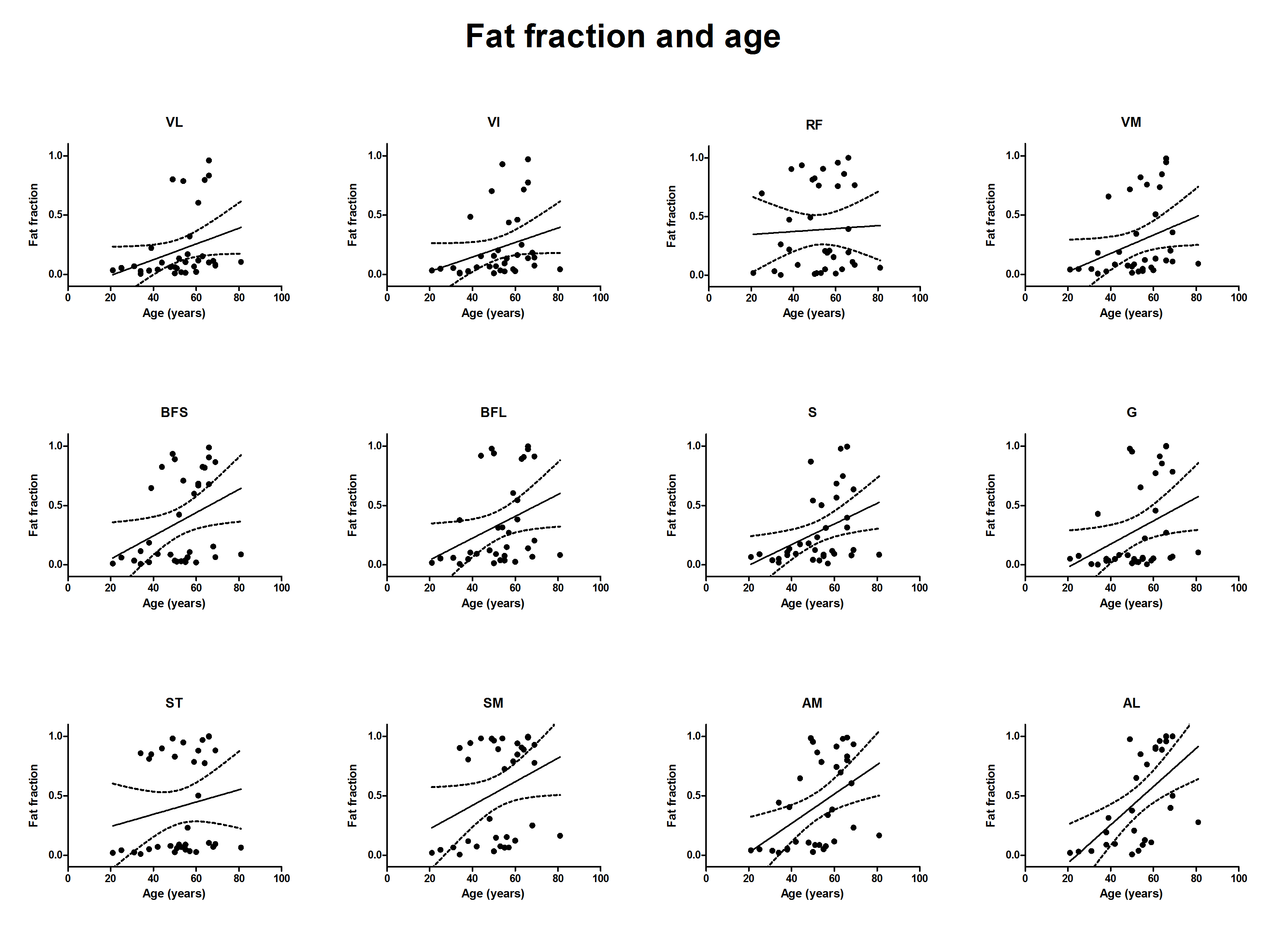

Supplement: Figure S1 — Correlation between fat fractions and age for the individual thigh muscles. Solid line gives best linear correlation with 95% confidence interval indicated by the dotted lines. Slopes of the lines were statistically tested to identify possible differences between the muscles. However analyses showed no significant differences. VL = vastus lateralis, VI = vastus intermedius, RF = rectus femoris, VM = vastus medialis, BFS biceps femoris short head, BFL = biceps femoris long head, S = sartorius, G = gracillis, ST = semit endinosus, SM = semi membranosus, AM = adductor magnus, AL = adductor longus. (TIF) [file pone.0085416.s001.tif]

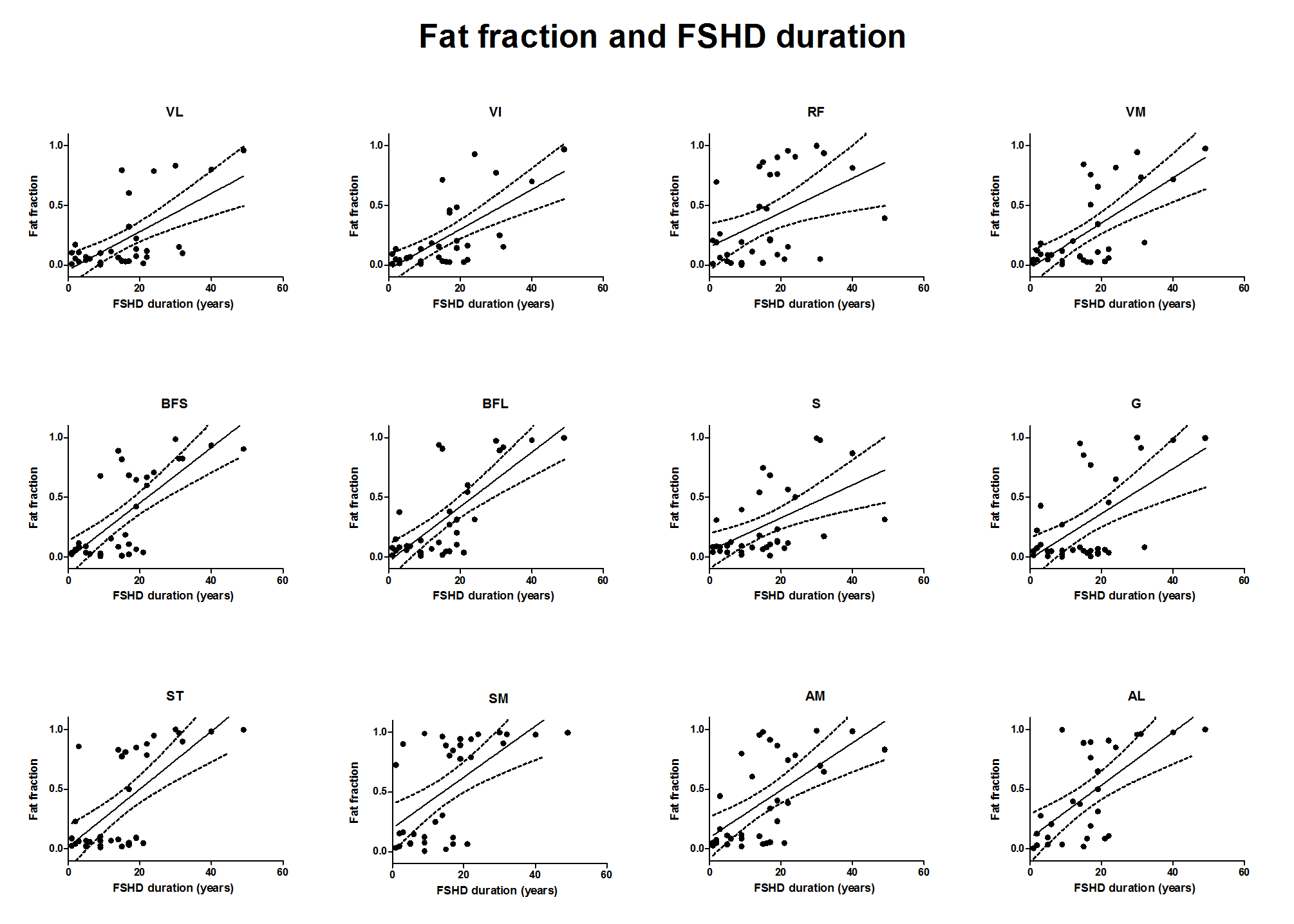

Supplement: Figure S2 — Correlation between fat fractions and disease duration for the individual thigh muscles. Solid line gives best linear correlation with 95% confidence interval indicated by the dotted lines. Slopes of the lines were statistically tested to identify possible differences between the muscles. However analyses showed no significant differences. VL = vastus lateralis, VI = vastus intermedius, RF = rectus femoris, VM = vastus medialis, BFS biceps femoris short head, BFL = biceps femoris long head, S = sartorius, G = gracillis, ST = semi tendinosus, SM = semi membranosus, AM = adductor magnus, AL = adductor longus. (TIF) [file pone.0085416.s002.tif]
